# *A*ustralasian *M*alignant *PL*eural *E*ffusion (AMPLE)-4 trial: study protocol for a multi-centre randomised trial of topical antibiotics prophylaxis for infections of indwelling pleural catheters

**DOI:** 10.1186/s13063-024-08065-1

**Published:** 2024-04-10

**Authors:** Estee P. M. Lau, Matthew Ing, Sona Vekaria, Ai Ling Tan, Chloe Charlesworth, Edward Fysh, Ranjan Shrestha, Elaine L. C. Yap, Nicola A. Smith, Benjamin C. H. Kwan, Tajalli Saghaie, Bapti Roy, John Goddard, Sanjeevan Muruganandan, Arash Badiei, Phan Nguyen, Mohamed Faisal Abdul Hamid, Vineeth George, Deirdre Fitzgerald, Nick Maskell, David Feller-Kopman, Kevin Murray, Aron Chakera, Y C Gary Lee

**Affiliations:** 1 Pleural Medicine Unit, Institute for Respiratory Health, Perth, Australia; 2 School of Medical and Health Sciences, Edith Cowan University, Perth, Australia; 3 Medical School, Faculty of Health & Medical Sciences, University of Western Australia, Perth, Australia; 4 Department of Respiratory Medicine, Sir Charles Gairdner Hospital, Perth, Australia; 5 Department of Pharmacy, Sir Charles Gairdner Hospital, Perth, Australia; 6 Department of Respiratory Medicine, St John of God Hospital Midland, Perth, Australia; 7 Curtin University Medical School, Perth, Australia; 8 Department of Respiratory Medicine, Fiona Stanley Hospital, Perth, Australia; 9 Department of Respiratory Medicine, Middlemore Hospital, Auckland, New Zealand; 10 Department of Respiratory Medicine, Wellington Regional Hospital, Wellington, New Zealand; 11 Department of Respiratory and Sleep Medicine, The Sutherland Hospital, Sydney, Australia; 12 University of New South Wales, Sydney, Australia; 13 Department of Respiratory Medicine, Concord Repatriation General Hospital, Concord, NSW Australia; 14 Faculty of Medicine and Health Sciences, Macquarie University, Sydney, Australia; 15 Department of Respiratory and Sleep Medicine, Westmead Hospital, Sydney, Australia; 16 Department of Respiratory Medicine, Sunshine Coast University Hospital, Birtinya, QLD Australia; 17 Griffith University, Brisbane, QLD Australia; 18 Department of Respiratory Medicine, Northern Health, Epping, VIC Australia; 19 Thoracic Medicine, Royal Adelaide Hospital, Adelaide, SA Australia; 20 Adelaide Medical School, Faculty of Health and Medical Science, University of Adelaide, Adelaide, SA Australia; 21 Respiratory Unit, Faculty of Medicine, Universiti Kebangsaan Malaysia, Kuala Lumpur, Malaysia; 22 Department of Respiratory and Sleep Medicine, John Hunter Hospital, New Lambton Heights, NSW Australia; 23 Hunter Medical Research Institute, Newcastle, Australia; 24 Department of Respiratory Medicine, Tallaght University Hospital, Dublin, Ireland; 25 Academic Respiratory Unit, Bristol Medical School, University of Bristol, Bristol, UK; 26 Department of Medicine, Section of Pulmonary and Critical Care Medicine, Dartmouth-Hitchcock Medical Center, Lebanon, NH USA; 27 School of Population and Global Health, University of Western Australia, Perth, Australia; 28 Renal Unit, Sir Charles Gairdner Hospital, Perth, Australia

**Keywords:** Mupirocin; Prophylaxis, Topical antibiotic, Indwelling catheter, Pleural, Infection

## Abstract

**Background:**

Malignant pleural effusion (MPE) is a debilitating condition as it commonly causes disabling breathlessness and impairs quality of life (QoL). Indwelling pleural catheter (IPC) offers an effective alternative for the management of MPE. However, IPC-related infections remain a significant concern and there are currently no long-term strategies for their prevention. The Australasian Malignant PLeural Effusion (AMPLE)-4 trial is a multicentre randomised trial that evaluates the use of topical mupirocin prophylaxis (*vs* no mupirocin) to reduce catheter-related infections in patients with MPE treated with an IPC.

**Methods:**

A pragmatic, multi-centre, open-labelled, randomised trial. Eligible patients with MPE and an IPC will be randomised 1:1 to either regular topical mupirocin prophylaxis or no mupirocin (standard care). For the interventional arm, topical mupirocin will be applied around the IPC exit-site after each drainage, at least twice weekly. Weekly follow-up via phone calls or in person will be conducted for up to 6 months. The primary outcome is the percentage of patients who develop an IPC-related (pleural, skin, or tract) infection between the time of catheter insertion and end of follow-up period. Secondary outcomes include analyses of infection (types and episodes), hospitalisation days, health economics, adverse events, and survival. Subject to interim analyses, the trial will recruit up to 418 participants.

**Discussion:**

Results from this trial will determine the efficacy of mupirocin prophylaxis in patients who require IPC for MPE. It will provide data on infection rates, microbiology, and potentially infection pathways associated with IPC-related infections.

**Ethics and dissemination:**

Sir Charles Gairdner and Osborne Park Health Care Group Human Research Ethics Committee has approved the study (RGS0000005920). Results will be published in peer-reviewed journals and presented at scientific conferences.

**Trial registration:**

Australia New Zealand Clinical Trial Registry ACTRN12623000253606. Registered on 9 March 2023.

**Supplementary Information:**

The online version contains supplementary material available at 10.1186/s13063-024-08065-1.

## Background

Malignant pleural effusion (MPE) is a significant healthcare problem [1]. It is a common complication of advanced cancer, most frequently in lung and breast cancers [2, 3], and its presence usually signifies incurable metastatic disease. The prognosis of patients with MPE is poor, with a median life expectancy ranging from 4 to 12 months [4], and the management of MPE remains palliative in nature. MPE causes disabling breathlessness that is debilitating and is associated with poor quality of life (QoL). Therefore, the primary goal of therapy is to provide effective control of symptoms with minimal interventions.

Indwelling pleural catheter (IPC) is an ambulatory drainage device for MPE patients that permits fluid evacuation at home, avoiding hospital visits. Multiple randomised studies have proven that IPC is significantly superior to conventional talc slurry pleurodesis for the management of MPE in reducing the need for further invasive pleural interventions and hospitalisation days while providing equivalent benefits in relieving breathlessness and QoL [5–7]. IPC is now established as one of the first-line management options for MPE in recent guidelines [8, 9].

Despite the advantages of IPC, there exists a 10–20% associated complication rate [10], with IPC-related infection being clinicians’ biggest hesitation in adopting IPC use. IPC-related infections usually develop after 6 weeks post-insertion [11] and include infections of the pleural cavity/fluid, catheter tract (i.e. tunnel infections), and skin at the exit-site (i.e. cellulitis). The incidences of IPC-related infections varied among studies and was reported to be as high as 25% [7]. The largest multi-centre study and a recent review of IPC-related pleural infections found *Staphylococcus aureus* as the most common causative organism [12, 13].

IPCs share many similarities with peritoneal dialysis (PD) catheters which are also frequently complicated by peritonitis and exit-site infections (ESIs). The use of topical antibiotics (especially mupirocin) to reduce PD-related infections have been a subject of several recent studies. Mupirocin is a topical antibiotic with excellent activity against gram-positive organisms and is an attractive prophylactic option for *S. aureus*-related infections [14].

Several studies demonstrated significant reduction in peritonitis and ESIs attributed to *S. aureus* or Gram-positive organisms when mupirocin was applied around the catheter exit site [15–19]. A meta-analysis [20] also found that mupirocin prophylaxis reduced the rate of *S. aureus* peritonitis and ESIs by 66% and 62% respectively. In a prospective controlled study [21], mupirocin reduced the overall incidence of peritonitis by 61% and ESI by 55%. Specifically, *S. aureus* peritonitis was cut by 100% and ESI by 65%. The International Society for Peritoneal Dialysis (ISPD) guidelines now recommend a daily application of topical mupirocin around the catheter exit-site as prophylaxis for peritoneal dialysis patients [22, 23].

To date, no long-term preventative approaches exist for IPC-related infections, which are significant events to cancer patients and delay oncologic treatments. Management of infection requires hospitalisations with associated costs. Having a prophylactic antibiotic to reduce/prevent infection is an attractive option, but whether the use of mupirocin prophylaxis can be extrapolated to IPC infections is unknown. Patients undergoing PD and those with MPE differ in underlying comorbidities and frequencies of fluid exchanges/drainages and are exposed to different sources of infection. Results of mupirocin use in PD cannot be directly extrapolated to MPE patients with an IPC without a randomised clinical trial. Our pilot study [24] established the safety and feasibility of mupirocin prophylaxis in patients with malignant effusions, and the AMPLE-4 trial represents the next step to investigate the efficacy.

## Methods

### Study design

The Australasian Malignant PLeural Effusion (AMPLE)-4 trial is a pragmatic, multi-centre, open-labelled, 1:1 randomised study to evaluate the use of regular prophylactic topical mupirocin (*vs* no mupirocin) to reduce catheter-related infections in patients fitted with an IPC for MPE.

### Study setting

**Fig. 1:**
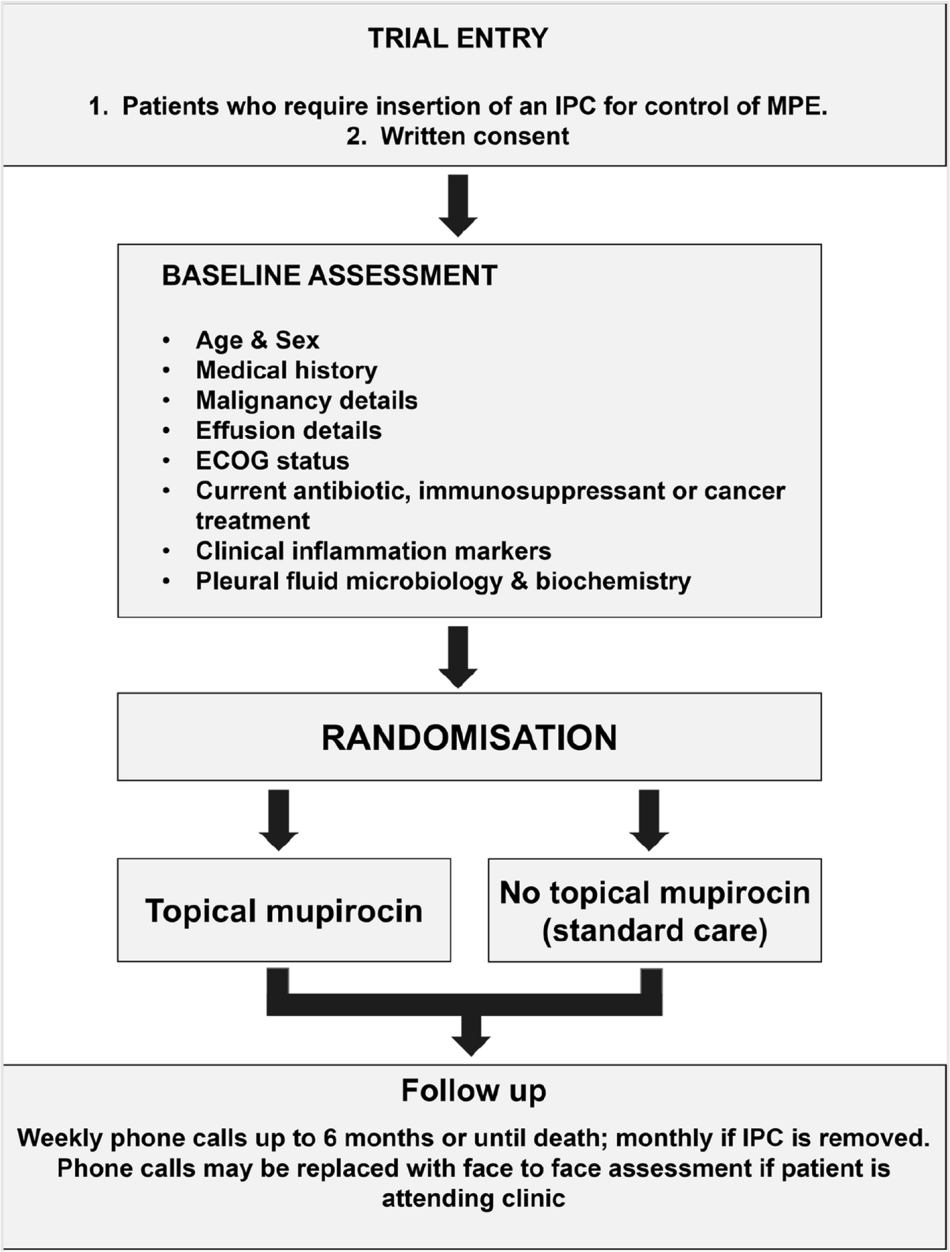
Study flow chart. ECOG, Eastern Cooperative Oncology Group; IPC, indwelling pleural catheter; MPE, malignant pleural effusion

This trial will be conducted at tertiary centres across Australia, New Zealand, and Asia, and new enrolling sites will be updated regularly on the trial registration website (URL: https://www.australianclinicaltrials.gov.au/anzctr/trial/ACTRN12623000253606). This trial will include 418 patients with MPEs, who require an IPC randomised 1:1 to either topical mupirocin or no topical mupirocin (standard care) (Fig. 1).

### Participant screening, selection, and recruitment

The site principal investigator or designated site research staff will screen patients with symptomatic MPE for when an IPC is planned for treatment. Potential participants will be approached about the study and be provided with the participant information and consent form to read and to ask questions to the study team. They will also be given time to discuss the study with family and carers and their general practitioner, if needed. Eligible participants will be offered trial entry and will be enrolled after providing informed consent. The site principal investigator will be aware of their dual role as the patients’ primary physician and as a clinical researcher and where this patient dependency can be a potential conflict. Enrolment and screening logs will be maintained.

### Inclusion criteria

Patients who require insertion of an IPC for control of MPE can be considered for inclusion. MPE is defined if cancer cells are identified in the pleural fluid or pleural biopsy or is a large exudative effusion without other causes in a patient with advanced disseminated malignancy.

### Exclusion criteria

Exclusion criteria includes age < 18 years, allergy to mupirocin, ipsilateral pleural infection within past 3 months, and inability to consent or comply with the protocol.

### Treatment

#### Topical mupirocin arm

For those assigned to the topical mupirocin (interventional) arm, topical mupirocin 2% (cream or ointment) will be applied around the exit-site of the IPC for an area approximately 3 cm in diameter. An information sheet with a picture of how to apply mupirocin will be provided to patients/carers. The antibiotic should be applied within 48 h of IPC insertion and thereafter following each drainage but at least twice weekly (with dressing change) until IPC is removed or the end of this study.

#### No topical mupirocin (standard care) arm

Patients assigned to the standard care arm will be managed in the conventional manner with the usual education and care of the IPC and without topical mupirocin prophylaxis.

### Clinical care

Participants in both arms will be managed by their own clinical teams and receive all other medical treatments (including chemotherapy and radiotherapy) as deemed clinically appropriate. Patients’ medical care, including IPC care and oncology management, will be directed by their attending physicians, as per standard practice in the treatment hospital, regardless of study group allocation. This includes the frequency of drainage, drainage device (suction bottle or drainage bag), and administration of talc pleurodesis via IPC. All patients will receive standard education on IPC aftercare, have access to support services (e.g. direct phone line), and receive usual care from their attending physicians. Decision of IPC removal is made by the physicians in-charge.

### Monitoring and follow-up

Potential participants, as part of the informed consent process, will have the study procedures and follow-up plan discussed in detail.

All patients (or their carers/nurses) will be contacted by phone every week to assess for clinical outcomes, compliance, or adverse events until death or end of 6-month follow-up period (Table 1). Frequency of the phone review will decrease to monthly once the IPC is removed. If the patient is attending hospital visits for other reasons, then the telephone review may be replaced by face-to-face assessment.

**Table 1:** Schedule of treatment for each visit and follow-up procedures

	**Study period**			
	**Pre-procedure**		**Post-procedure**	
	**Enrolment**	**Index procedure**	**Days post-procedure**	**Week**^**a**^
**Timepoint**	***Baseline***	***D0***	***D1* ± *24 h***	***1–24***
**Enrolment:**				
**Informed consent**	X			
**Baseline data collection**	X			
**Randomisation**	X			
**IPC insertion**		X		
**Interventions**				
**Dressing change ± mupirocin application**			X	X
**Assessments:**				
**Phone call/clinic visit**				X
**Adverse event review**			X	X
**Evaluation of exit-site**			X	X

^a^Weekly follow-up for up to 6 months (monthly if IPC is removed); *IPC*, indwelling pleural catheter

Where participants do not answer follow-up calls/attend planned study visits, the research staff will contact them again or book an additional visit if required. If the patient is an inpatient, the visit will be carried out in the hospital, if appropriate.

### Outcomes

Data on primary and secondary endpoints will be captured weekly (monthly if IPC is removed) from catheter insertion until death or end of 6-month follow-up period. Outcomes will be reported as mean or median, as appropriate.

### Primary endpoint

The primary outcome is the percentage of patients who developed an IPC-related infection from catheter insertion until death or end of 6-month follow-up period. IPC-related infection can be any one of the following:

- Pleural infection: presence of pus and/or bacteria (by Gram stain or culture) in pleural fluid plus a clinical picture compatible with infection (e.g. fever, leucocytosis, raised inflammatory markers).
- Catheter tract infection: signs of inflammation along the tract usually with swelling and significant tenderness plus a clinical presentation compatible with infection.
- Cellulitis at exit-site: signs of inflammation clinically warranting systemic antibiotic treatment as determined by the attending physician.

### Secondary endpoints

- Infection will be analysed:
  As the total number of episodes for all patients in each group;
  As percentage of patients and as total number of episodes—each adjusted for number of days IPC is in situ for each patient;
  As each of the individual types of infection;
  Time to first episode of infection; and
  For organism(s) causing infection (e.g. *S. aureus vs *others)
- Hospital days will be analysed:
  As total days in hospital (for any reasons)
  As days related to IPC-related infections, similar to methods used in prior AMPLE trials [6, 25]. All records of hospitalisation will be reviewed by an independent investigator.
- Adverse and serious adverse events will be recorded as in previous AMPLE trials [6, 25]. Definitions for adverse and serious adverse events are listed under adverse events section in the protocol.
- Resources used associated with antibiotics use and IPC-related infections will be obtained from discharge letters and hospital in-patient enquiry coding. In-/out-patient management of any related complications will be captured from hospital records or self-reports from patients and will include treatments, imaging, and other interventions related to the adverse events. An experienced health economist will oversee this study aspect.
- Survival will be measured from randomisation to death or end of study follow-up.

### Sample size

This study will enrol 418 patients to detect a difference in IPC-related infection rate between the treatment arms. The difference that we wish to detect is 10% in the topical mupirocin prophylaxis arm (i.e. a relative reduction in infection rates of 50%) *vs* 20% in the no topical mupirocin prophylaxis (standard care) arm (based on previous studies) [7, 15–18, 25]. Previous randomised clinical trials (RCTs) reported a pleural infection rate of ~ 10% [7, 25]. Incidences of tract infection and cellulitis (combined) are often similar to the pleural infection rates in published papers. Hence, we estimated a 20% incidence for overall IPC-related infections. In the RCTs investigating mupirocin prophylaxis in PD patients, a two-third reduction in infection rates (*vs* control arms) were commonly reported [15–17]. To be conservative, we therefore estimated an incidence of 10% in our treatment arm. The sample size calculation was carried out using an anticipated chi square test to compare these proportions, assuming a 5% significance level and a power of 80%. To achieve this, we would need 199 patients per group (with an additional 5% to allow for dropouts based on previous AMPLE trial [6]), giving a total of 418 patients.

### Randomisation

Participants will be randomised 1:1 to either topical mupirocin or no topical mupirocin (standard care). Randomisation will include minimisation for (i) cancer type (mesothelioma *vs* non-mesothelioma), (ii) known presence of trapped lung (*vs* not), (iii) ECOG performance status (≤ 2 *vs* ≥ 3), and (iv) current immunosuppression (or chemotherapy) *vs* not. The Griffith Randomisation Service by the Griffith University, Queensland, Australia, provides the randomisation setup via their automated web portal. The site principal investigator or designated site research staff screening patients will be able to generate the allocation sequence using the automated centralised randomisation system, enrol participants, and assign participants to interventions based on the randomisation.

### Data management and safety

All procedures for the handling and analysis of data will be conducted using GCP ICH guidelines and the National Statement on Ethical Conduct in Human Research (2007) – *Updated 2018* and in accordance with local policies and procedures.

Patient privacy and confidentiality will be maintained, as any information that identifies participants will be available only at the enrolment study site and only to designated study investigators, all of whom will either have signed a confidentiality agreement or be employees of the hospital.

Data collected will be stored in line with the Australian Code for the Responsible Conduct of Research for clinical trials and local policy guidelines for research data archiving. Access to the final trial dataset will only be available to the research team at the lead site.

Audits, if any, are usually carried out by an independent compliance monitoring officer.

### Statistical plan

Data will be analysed on an intention-to-treat basis and per protocol basis. All participants, excluding those who withdrew prior to the randomisation intervention, will be included in the intention-to-treat analyses and analysed according to their randomised assignment. Per-protocol analyses will be performed in participants who have had at least one week of mupirocin application *vs* those who did not have any. Sensitivity analysis, e.g. with multiple imputation, will be carried out whenever appropriate, to account for missing data. The primary outcome will be analysed using chi-square test and subsequent logistic regression analyses allowing adjustments for minimisation variables. A secondary analysis of the primary outcome will utilise the time to event data, where cumulative incidence plots will be presented, and the log-rank statistic used to compare the treatment groups. In addition, Cox regression models will be used to calculate cause specific hazard ratios adjusted for minimisation variables. A competing risk analysis will also be performed to account for the competing risk of death in estimation of event rates. For binary or continuous secondary outcomes, inter-group differences will be examined using chi-square tests or two sample *t*-tests respectively, with additional logistic and linear regression analyses adjusting for minimisation variables. Adverse and serious adverse events will be reported in descriptive figures. Data analysts will be masked to the assigned groups, where appropriate.

An interim analysis is planned after 100 patients have been enrolled and completed follow-up. The purpose is to (i) assess the rate of recruitment and determine the feasibility of fulfilling the enrolment target and (ii) futility—observe the actual incidence of event rates in the control group to ensure the study is adequately powered to detect a clinically meaningful difference. We would determine if the conditional power based on the trend observed at the interim analysis decreases to less than 0.2. The need for further interim analysis will be assessed accordingly, and any decision to terminate the trial will be made by the trial steering committee.

### Ethics

The trial has been approved (as of 20 December 2023) by the following committees:

1. Sir Charles Gairdner Osborne Park Healthcare Group Human Research Ethics Committee (HREC) for Australian public hospitals, Australia
2. St John of God Health Care Ethics Committee for Midland Hospital, Western Australia
3. Universiti Kebangsaan Malaysia Research Ethics Committee for University Kebangsaan Malaysia Medical Centre, Malaysia
4. Northern B Health and Disability Ethics Committee for hospitals in New Zealand
5. Macquarie University Human Research Ethics Committee, Medical Sciences, Australia

Study investigators will ensure that any amendments to the protocol is approved by the ethics committee and signed by any patients subsequently entering into the trial and those currently in the study, if affected by the amendment.

### Trial monitoring and oversight

The trial steering committee (TSC) will be responsible for the supervision of the trial in all its aspects. It will be responsible for ensuring the completion of the trial to clinical and ethical standards. Members of the TSC include an independent chairperson, independent member(s), chief investigator and selected investigators, a consumer representative, and the trial coordinator(s). The TSC will monitor site recruitment and review any recommendations received from the data and safety monitoring board (DSMB). The DSMB ensures the safety of study participants through monitoring of ethical conduct of the study and study procedures, reviewing adverse events, and considering new data (recently published studies) that may determine the validity of study continuation. The DSMB includes an independent chairperson and other independent members, one of whom is a statistician.

### Sponsorship

The study is sponsored by the Institute for Respiratory Health, a not-for-profit organisation. Contact details: Mr Bi Lam, Finance Manager, Level 2, 6 Verdun Street, Nedlands WA 6009.

t|+ 61 8 6151 0877 e| bi.lam@resphealth.uwa.edu.au.

### Adverse events

All adverse events relating to the study, serious and non-serious, will be fully documented according to the ‘Adverse Event Reporting’ Section of the Investigator Site File. An adverse event is defined as any untoward medical occurrence, including an exacerbation of a pre-existing condition in a patient in a clinical investigation who received an experimental procedure. The event does not necessarily have to have a causal relationship with this treatment. A serious adverse event is defined as any adverse event/adverse reaction that results in death, is life-threatening, requires hospitalisation or prolongation of existing hospitalisation, and results in persistent or significant disability of incapacity.

All adverse events relating to and occurring during the course of the clinical study (i.e. from signing the informed consent until death or the end of the study follow-up period, whichever comes first) will be collected, documented, and reported to the DSMB. Events will also be reported if a causal link (relatedness) between the adverse event and the study is suspected but not confirmed.

### Plans for dissemination

Results from this study will be published in peer-reviewed journals and presented at national and international conferences. Authorship eligibility guidelines will be discussed during the TSC meetings.

## Discussion

This is the first randomised trial to investigate a long-term preventative strategy for IPC-related infections. It is designed to be pragmatic, with few exclusion criteria to ensure that the results are generalisable. Whether the benefits of topical mupirocin prophylaxis in the context of peritoneal dialysis can be extrapolated to IPC for MPE patients requires robust examination. The presence of a control group in AMPLE-4 will provide insight into the potential efficacy of mupirocin prophylaxis in reducing IPC-related infections in patients with MPE.

The primary outcome of AMPLE-4, which includes all three types of IPC-related infections (pleural, tract and skin), will provide a more comprehensive understanding of the actual IPC-related infection rate, as existing literature mainly focussed on reporting IPC-related pleural infections, with catheter tract infections and cellulitis being less well documented. Furthermore, the findings from this study will enhance our understanding of the microbiology and potentially infection pathways associated with IPC-related infections, bridging knowledge gaps which are crucial for advancing the field.

### Trial status

Protocol version: Version 2.00/16.10.23.

Date recruitment began: 21.07.23.

Estimated recruitment completion date: end of March 2026.

### Supplementary Information

**Supplementary Material 1.**

## Data Availability

Investigators at the lead site will have access to the final trial dataset. Supporting data including standard operating procedures, details of data management procedures, case report forms, and datasets generated and/or analysed during the current study will be available to the scientific community with as few restrictions as possible, while retaining exclusive use until publication of major outcomes. Data requests from qualified researchers should be made to YCGL (gary.lee@uwa.edu.au).

## Abbreviations

ESI: Exit-site infection
DSMB: Data and safety monitoring board
IPC: Indwelling pleural catheter
ISPD: International Society for Peritoneal Dialysis
MPE: Malignant pleural effusion
PD: Peritoneal dialysis
QoL: Quality of life
TSC: Trial steering committee
